# Clay-supported bio-based Lewis acid ionic liquid as a potent catalyst for the dehydration of fructose to 5-hydroxymthylfurfural

**DOI:** 10.1038/s41598-023-50773-2

**Published:** 2024-01-02

**Authors:** Soheila Yaghoubi, Samahe Sadjadi, Xuemin Zhong, Peng Yuan, Majid M. Heravi

**Affiliations:** 1 Department of Chemistry, School of Physics and Chemistry, Alzahra University, Vanak, PO Box 1993891176, Tehran, Iran; 2 Gas Conversion Department, Faculty of Petrochemicals, Iran Polymer and Petrochemical Institute, PO Box 14975-112, Tehran, Iran; 3 CAS Key Laboratory of Mineralogy and Metallogeny/Guangdong Provincial Key Laboratory of Mineral Physics and Materials, Guangzhou Institute of Geochemistry, Chinese Academy of Sciences, Guangzhou, 510640 China; 4 School of Environmental Science and Engineering, Guangdong University of Technology, Guangzhou, 510006 China

**Keywords:** Chemistry, Catalysis, Heterogeneous catalysis

## Abstract

Caffeine and halloysite nanoclay mineral that are bio-based compounds were utilized to synthesize a novel Lewis acid heterogeneous catalyst. To this aim, halloysite was functionalized with 2,4,6-trichloro-1,3,5-triazine and reacted with caffeine, which was then converted to ionic liquid via a reaction with ZnCl_2_. The catalyst was applied for promoting the dehydration of fructose to 5-hydroxymethylfurfural. To investigate the effects of the reaction variables, response surface methodology was used. The product was achieved in 98.5% in 100 min using a catalyst loading of 30 wt% at 100 °C. Moreover, the catalyst was recyclable up to six runs with slight zinc leaching. Comparison of the catalytic activity of the catalyst with that of halloysite and a control catalyst with one caffeine-based Lewis acid ionic liquid confirmed the superior activity of the former and the important role of 2,4,6-trichloro-1,3,5-triazine for increasing the number of the grafted caffeine and thus the acidic sites of the catalyst. A plausible reaction mechanism was proposed, and the activity of the catalyst for other carbohydrates was also studied. According to the results, this catalyst catalyzed the reaction of other substrates to furnish 5-hydroxymethylfurfural in low to moderate yields. According to the kinetic studies, the activation energy was estimated to be 22.85 kJ/mol.

## Introduction

Increasing use of fossil fuels resulted in the emission of huge amounts of carbon dioxide, methane and other pollutants into the atmosphere^1^. This issue caused considerable threat for both human health and ecosystem^2,3^. To furnish a solution to these concerns, sustainable development has gained remarkable attention. In this line, use of renewable resources, such as biomass for energy production is on the agenda^2^. Conversion of various types of biomass, such as agricultural waste, plants, wood, etc. can result in electricity and fuel, denoted as biofuel^4^. This clean and accessible energy can be considered as a potent alternative to the conventional fossil fuels^5^. In this context, development of furan-based biofuels, which have high energy densities and can be obtained from non-edible resources is considered as a breakthrough^6,7^. One of the most promising furan-based biofuels is 2,5-dimethylfuran that can be obtained from hydrodeoxygenation of 5-hydroxymethylfurfural (HMF). In fact, HMF, which contains two functional groups of aldehyde and alcohol^8^ is a versatile compound that can be applied for the synthesis of not only biofuels, but also other chemicals, such as levulinic acid, 2,5-diformylfuran, and 2,5-bis(ethoxymethyl)furan^9,10^. This key furan compound can be synthesized from various carbohydrates, such as fructose, galactose etc. under acidic condition. The most important challenge for HMF synthesis is its instability and susceptibility to form side-products, including levulinic acid and formic acid as well as cross-polymerization and formation of humins. To circumvent this issue, wise choice of the solvent^11^ and tuning the acidity of the acidic catalysts^12^ as well as optimization of the reaction conditions are inevitable. To date, numerous acidic catalysts, such as Lewis acid catalysts^13^, phosphate based catalysts, such as titanium and zirconium phosphates^14^, heteropolyacids^15,16^, metal–organic frameworks-based catalysts^17^, functionalized silica-based catalysts^18^ and H-shaped zeolites^19^ have been introduced for this acid-catalyzed reaction, among them, ionic liquids (ILs)^20^ are one of the suitable candidates for the synthesis of HMF. ILs, first synthesized by Paul Walden in 1914, consist of a large and bulky cationic organic moiety and a smaller and anionic organic or non-organic part. These organic salts benefit from unique properties, including thermal and mechanical stability, low vapour pressure and tunable physical and chemical properties through adjusting their cations and anions^21–23^. These outstanding features render ILs potent candidates for various uses, such as catalysis, supercapacitors, batteries etc. ILs were successfully employed for HMF synthesis^24^. Notably, most of ILs are synthesized from heterocyclic cations, such as imidazolium and pyridinium moieties^25^. This issue not only increase the cost of synthetic procedure, but also leads to the formation of less-environmentally benign catalysts.

Another drawbacks of ILs is their homogeneous nature, which makes their recovery and reuse tedious. As a solution to this challenge, ILs can be immobilized on supports, such as clays to form heterogeneous catalysts with improved recovery ad recyclability. Clays as natural compounds are of high priority as supporting materials. Various clays with different chemical compositions and morphologies are available in large scales. Hence, a suitable clay can be selected for a specific catalytic purpose^26,27^. In this regard, halloysite nanoclay (Hal) that is an aluminosilicate (Al_2_(OH)_4_Si_2_O_5_·2H_2_O) with cylindrical morphology^28–35^ has receive considerable uses in the catalysis^36^.

**Scheme 1:**
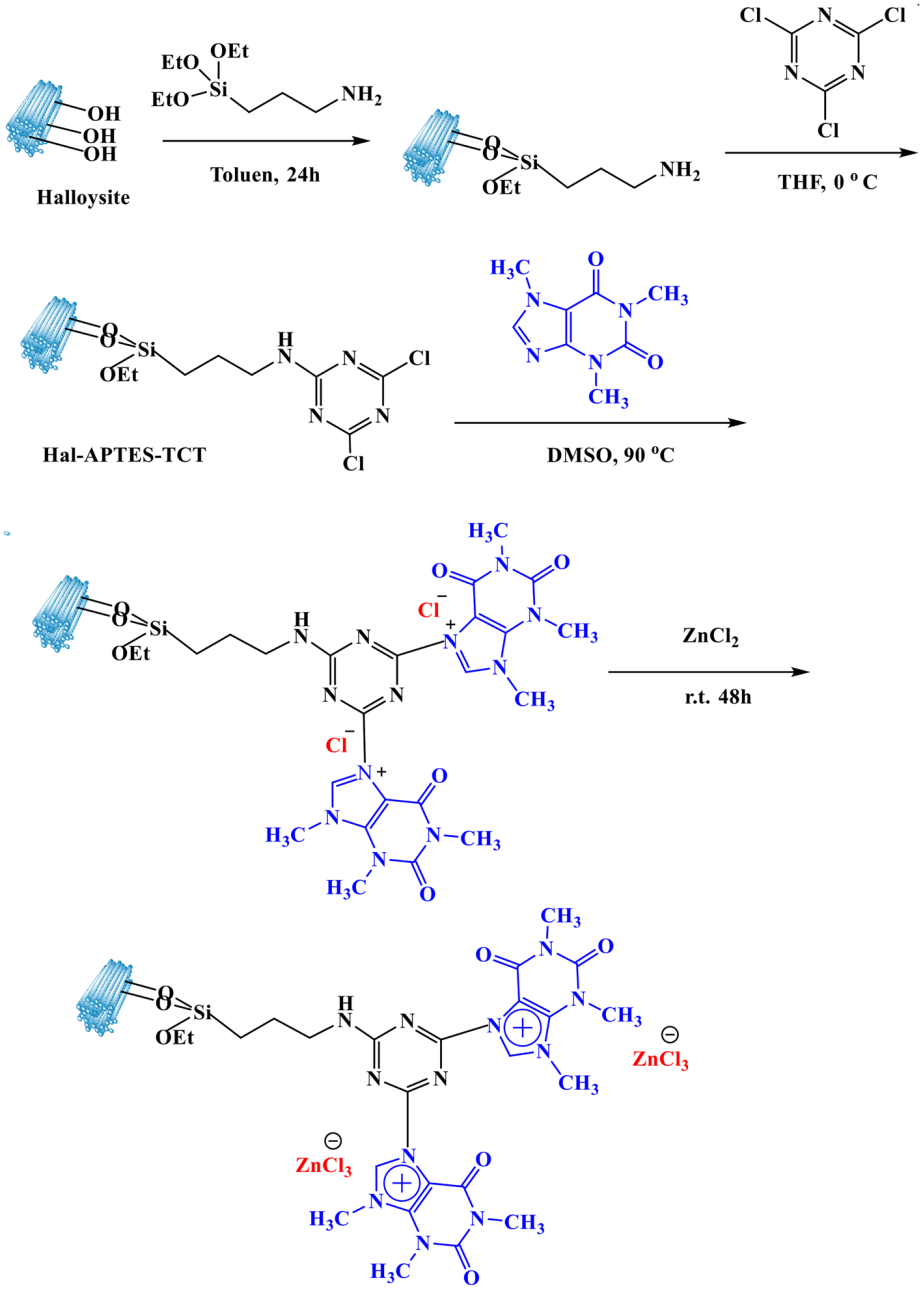
Pictorial procedure for the synthesis of Hal-IL.

In this research, using caffeine as a bio-based compound and halloysite as a naturally occurring clay mineral, a novel bio-based heterogeneous Lewis acid IL is designed and synthesized, Scheme 1. The reason for use of Hal as a support was its general features as a natural clay, such as high thermal, mechanical and chemical stability, as well as its unique tubular morphology. Indeed, our previous works on Hal-based catalysts^36^ disclosed that Hal shows excellent catalytic performance. On the other hand, studies on the IL-based catalysts for fructose conversion^37^ showed that imidazolium-based IL are more effective. Hence, we decided to use a bio-based imidazole source, caffeine, instead of conventional imidazoles. In fact, use of caffeine can decrease the cost of synthesis of the catalyst and lead to more environmentally-benign protocol for the preparation of the catalyst. To the best of our knowledge it is the first time that halloysite nanoclay is functionalized with caffeine-based IL. Moreover, use of 2,4,6-trichloro-1,3,5-triazine in the structure of the catalyst allows increase of the number of IL and consequently the acidity of the final catalyst. The as-prepared Hal-supported caffeine Lewis acid IL was then characterized and applied as an acidic catalyst for promoting conversion of fructose to HMF. Using Response surface method (RSM), the reaction conditions were optimized and the effects of the reaction variables on the yield of HMF were studied. The recyclability of the catalyst as well as its structural stability upon recycling were also confirmed.

## Result and discussion

### Hal-IL structure

**Figure 1:**
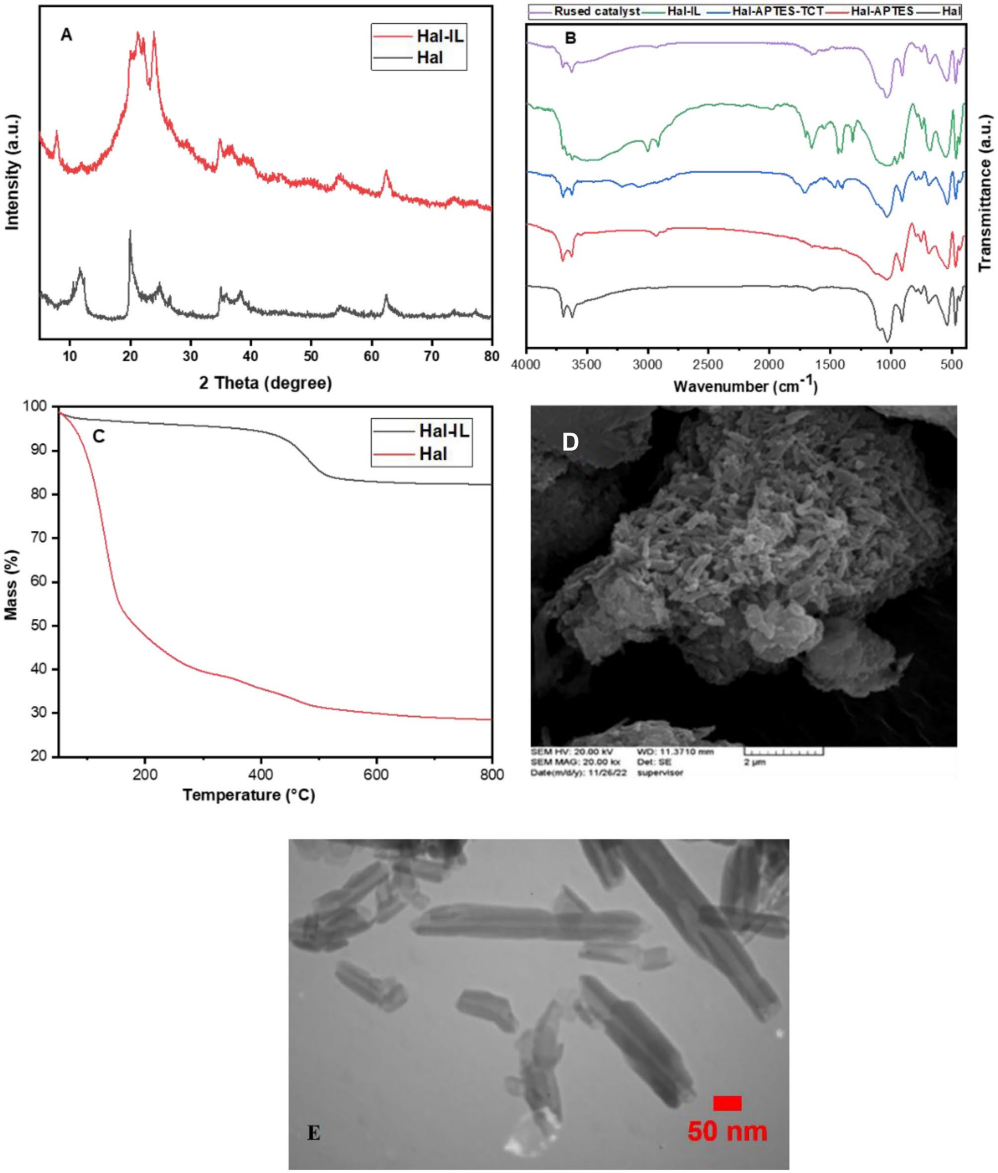
(**A**) XRD patterns of the catalyst (Hal-IL) and Hal, (**B**) FTIR spectra of Hal, Hal-APTES, Hal-APTES-TCT, the catalyst, and the reused catalyst, (**C**) TG curves of the catalyst (Hal-IL) and Hal, (**D**) SEM image of Hal-IL, (**E**) TEM image of the catalyst.

In Fig. 1A the XRD patterns of Hal and Hal-IL are shown and compared. As depicted, the Hal provided from China exhibited the peaks at 2θ = 12.3°, 18.8°, 20.6°, 25.2°, 35.7°, 38.1°, 56.3° and 62.5°. Although in the XRD pattern of Hal-IL, the characteristic peaks of Hal are observed, this pattern is distinguished from pristine Hal. In this XRD pattern, the broad band in the range of 2θ = 16.3°, 30.1°, which contains some Hal peaks is ascribed to the amorphous caffeine^38^. According to the literature, it is expected that characteristic peaks of ZnCl_3_^−^/ZnCl_4_^−^ appear at 2θ = 36.6°, 38.7°^39–41^ that overlapped with the Hal peaks. The comparison of the two XRD patterns, not only confirms IL conjugation, but also approves stability of Hal upon functionalization with IL.

To confirm formation of the desired product in each step of synthesis of the catalyst, FTIR spectra of Hal, Hal-APTES, Hal-APTES-TCT and Hal-IL were compared, Fig. 1B. As known, Hal FTIR spectrum showed the absorbance bands at 535 cm^−1^ (Al–O–Si vibration), 747 cm^−1^ (stretching vibration of Si–O), 795 cm^−1^ (symmetric stretching of Si–O), 1034 cm^−1^ (Si–O stretching), 1101 cm^−1^ (perpendicular Si–O–Si stretching), 1659 cm^−1^ (weak stretching and bending vibrations of water), 3696 cm^−1^ and 3625 cm^−1^ (interior –OH). These characteristic bands are detectable in the FTIR spectra of Hal-APTES, Hal-APTES-TCT and Hal-IL, indicating the structural stability of Hal in each synthetic step. In the FTIR spectrum of Hal-APTES, the absorbance band at 2968 cm^−1^ is indicative of –CH_2_ functionality of APTES, approving its successful attachment on Hal. In the case of Hal-APTES-TCT, the band appeared at 1714 cm^−1^ is attributed to the –C=N functionality, affirming incorporation of TCT in the structure of the catalyst. Comparison of the FTIR spectrum of Hal-IL with the other spectra implied that apart from the characteristic absorbance bands of Hal, APTES and TCT, the band at 1708 cm^−1^ can be observed that is ascribed to the amidic –C=O functionality. Notably, the stretching peak of Zn-Cl is expected at 510 cm^−1^, which overlapped with the bands of Hal^38^.

TGA was also employed to study the thermal behavior of the catalyst and affirm conjugation of IL on Hal. As shown in Fig. 1C, Hal is a thermally stable clay with weight losses due to the loss of water and dehydroxylation (~ 500 °C). Hal-IL thermogarm differs from that of Hal and exhibits a significant weight loss (51 wt%) in the range of 150–300 °C, which is due to the decomposition of IL.

Morphological study of Hal-IL, Fig. 1D, confirms that Hal tubular morphology is preserved upon incorporation of IL. Furthermore, aggregation of Hal tubes can be ascribed to the presence of IL and possible electrostatic interactions between charged moieties. Moreover, the TEM image of the catalyst, Fig. 1E, established that the catalyst showed tubular morphology.

To further approve incorporation of IL on Hal, EDS analysis, Fig. S1, was conducted. As depicted, Al, Si, O, N, C, Cl and Zn atoms are present in Hal-IL. Observation of Zn, Cl, C and N is a proof for the presence of IL in the structure of the catalyst. Elemental mapping analysis, Fig. S1, was also performed to give an insight onto the dispersion of functional group on Hal. As displayed, the representative atoms of IL showed relatively homogeneous dispersion, indicating that IL was formed all over Hal tubes.

Measurement of the specific surface area of Hal-IL and its comparison with that of Hal indicated that upon grafting of IL on Hal, this value decreased from 48 to 23 m^2^/g. this result approved immobilization of IL on Hal. In Fig. S2, nitrogen adsorption–desorption isotherm of Hal-IL is presented. According to the IUPAC classification, this isotherm showed type II with H3 hysteresis loops.

Among various methods, used for the measurement of the catalyst acidity^42^, NH_3_-TPD method was utilized for the investigation of the acidic features of the catalyst. The results indicated that Hal-IL possesses both weak and strong acidic sites and its total acidity was estimated to be 2336 micro mol/g cat.

### Catalytic performance of Hal-IL for HMF production

#### Catalytic activity

To achieve maximum yield of HMF, effective parameters on the reaction yield, i.e. reaction time, temperature and Hal-IL loading were optimized using RSM. The outcomes of analysis of variance (ANOVA) applying a quadratic model for the aforesaid parameters are listed in Table 1.

**Table 1:** ANOVA results of response surface method using quadratic model.

Source	Sum of squares	df	Mean square	F-value	P-value
Model	7477.36	9	830.82	33.76	< 0.0001
A—Temperature	367.78	1	397.78	14.95	0.0031
B—Time	462.79	1	462.79	18.81	0.0015
C—Catalyst	603.32	1	603.32	24.52	0.0006
AB	36.59	1	36.59	1.49	0.2506
AC	105.92	1	105.92	4.30	0.0648
BC	1294.13	1	1294.13	52.59	< 0.0001
A^2^	247.95	1	247.95	10.08	0.0099
B^2^	1180.74	1	1180.74	47.98	< 0.0001
C^2^	4161.57	1	4161.57	169.12	< 0.0001

The equation derived from the coded factors is as follow (Eq. 1):

1 $${\text{HMF Yield }}\left( \% \right) \, = \, + {1}00.{27} + {4}.{\text{79 A}} - {5}.{\text{38 B}} + { 6}.{\text{14 C }} + { 2}.{\text{14 AB}} - { 3}.{\text{64 AC}} - {12}.{\text{72 BC}} - { 3}.{\text{14 A}}^{{2}} - {6}.{\text{85 B}}^{{2}} - {12}.{\text{87 C}}^{{2}} .$$

A: Temperature, B: Time, C: Catalyst amount.

As described in Eq. (1), the sign of A, C, B and AB are positive, while the sign of B, AC, BC, A^2^, B^2^ and C^2^ are negative. In the RSM, the positive parameters have synergistic effect on the HMF yield (response), while the negative parameters have antagonistic effect. In this equation, the coefficients of the parameters indicate their significance. Hence, it can be concluded that C^2^ and BC have the most pronounced effects, while the effect of AB is the lowest.

The values of the correlation coefficient, R^2^, the Adjusted R^2^ and The Predicted R^2^ were estimated to be 0.968, 0.939 and 0.742 respectively.

**Figure 2:**
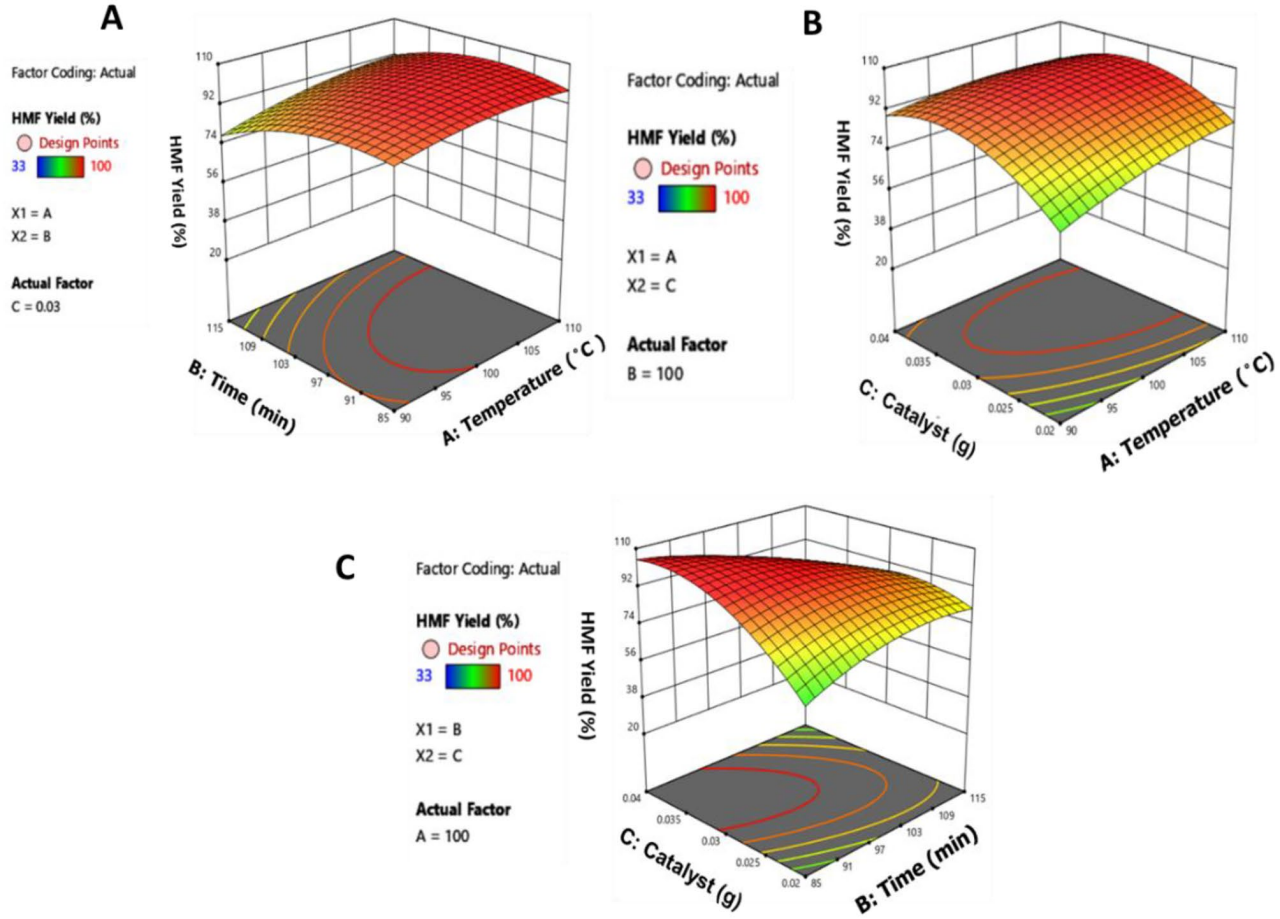
(**A**) 3D surface plot of the interaction between temperature and time for HMF yield, (**B**) 3D surface plot of the interaction between temperature and Hal-IL amount for HMF yield, (**C**) 3D surface plot of the interaction between time and Hal-IL amount for HMF yield.

3-D graphs of HMF yield (response) versus various reaction parameters (temperature, time and Hal-IL loading) are illustrated in Fig. 2.

3D surface plot of the interaction between temperature and time for HMF yield, Fig. 2A, indicates that by increase of the reaction temperature and time to 100 °C and 100 min, the maximum yield of HMF was achieved.

The results from 3D surface plot of the interaction between temperature and Hal-IL loading for HMF yield, Fig. 2B, imply that increment of the catalyst loading to 0.03 g led to the highest yield of HMF. Furthermore, the optimum value for reaction temperature was obtained as 100 °C.

Figure 2C shows 3D surface plot of the interaction between time and catalyst amount for HMF yield. Considering this plot, it can be concluded that using 0.03 g Hal-IL led to the highest yield of HMF after 100 min.

According to RSM results, the optimum reaction conditions were using 0.03 g Hal-IL at 100 °C in 100 min.

#### Control catalyst

In this study to design an acidic bio-based catalyst for promoting conversion of fructose to HMF, Hal, which is a natural clay mineral was used a support. Caffeine, on the other hand, that is a natural compound with imidazole in its structure was covalently conjugated on Hal and then converted to Lewis acid through reaction with ZnCl_2_. In the synthetic procedure, to increase the acidity, the number of grafted caffeine was theoretically doubled by using TCT, Scheme 1. To appraise whether the grafted Lewis acid could improve the catalytic activity of pristine Hal, dehydration of fructose was accomplished under the optimal conditions in the presence of pristine Hal. Interestingly, it was revealed that Hal showed low catalytic activity and led to the formation of HMF in 15% yield, Table 2. This is because of the acidity of Hal. Next, to investigate the role of number of the conjugated Lewis acid IL in the catalytic activity of the final catalyst, a control catalyst, Hal-IL1 (Scheme S1), was synthesized. As TCT was not used for the synthesis of Hal-IL1, it was expected that the grafted caffeine-based IL was less than that of Hal-IL. Notably, Hal-IL1 catalyst could catalyze dehydration of fructose to HMF to give 54% yield. More accurately, the catalytic activity of Hal-IL1 was higher than that of Hal and lower than that of Hal-IL. This result confirmed the role of TCT in increasing the acidic sites.

**Table 2:** Comparison of efficiency of Hal-IL with control catalysts for dehydration of fructose to HMF.

Entry	Catalyst	HMF yield (%)
1	Hal	15
2	Hal-IL-1	54%
3	Hal-IL	98.5%

Reaction conditions: catalyst loading 30 wt%, temperature 100 °C, time 100 min.

#### Recyclability

**Figure 3:**
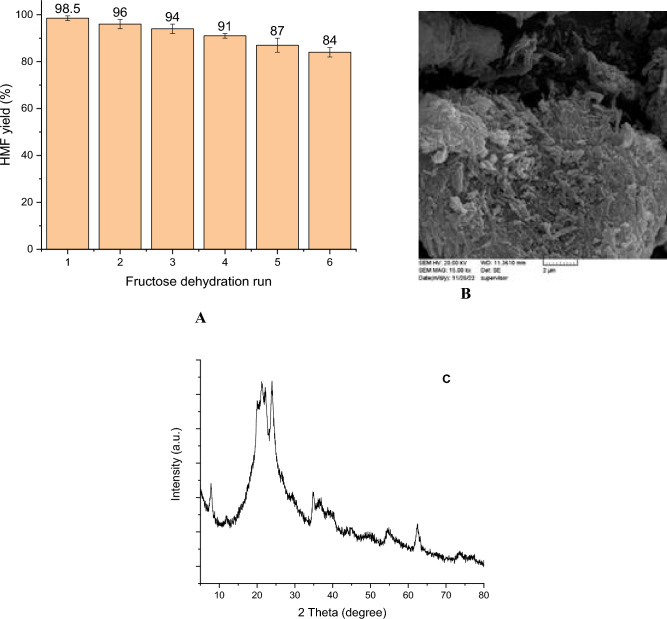
(**A**) Recyclability of Hal-IL for the conversion of fructose to HMF under the optimized conditions, (**B**) SEM image of Hal-IL after the last run of recycling and (**C**) XRD pattern of the recycled catalyst.

To study the recyclability of Hal-IL for the conversion of fructose to HMF, the recovered catalyst at the end of the first run of fructose dehydration was used again in the second run of the same reaction under the optimized conditions. This recovery-reuse cycle was continued up to six consecutive dehydration runs. As illustrated in Fig. 3A, the results affirmed high recyclability of Hal-IL. More precisely, each run of reuse led to only slight loss of the activity of the catalyst. These results approve to the stability of Hal-IL. In other word, as the acidic IL is attached on Hal covalently, it won’t be dissociated upon recovery and recycling. To confirm this issue, FTIR spectrum of the reused catalyst after the last run of fructose dehydration was recorded, Fig. 1B. The comparison of the FTIR spectra of the fresh and reused Hal-IL indicates the similarity of the two spectra, confirming the structural stability of the catalyst. To further study the stability of the catalyst in the course of recycling, ICP analysis was conducted to measure leaching of zinc species. According to the results, upon each run of recycling a light leaching of zinc species occurred and upon the last run of recycling the loading of zinc species decreased by 1.4%. This issue can justify the observed loss of activity upon each recycling run. Also, to investigate the structural stability of the recycled catalyst, its XRD pattern was recorded. As displayed in Fig. 3C, the XRD pattern of the recycled catalyst is similar to that of the fresh ones and exhibited all of the characteristic peaks of Hal-IL with no displacement, underlining the stability of the recycled Hal-IL.

Further characterization of the recovered Hal-IL after the last run of recycling was conducted using SEM analysis. As depicted in Fig. 3B, the morphology of Hal-IL after six runs is similar to that of fresh catalyst, implying the stability of Hal-IL.

#### Generality

**Figure 4:**
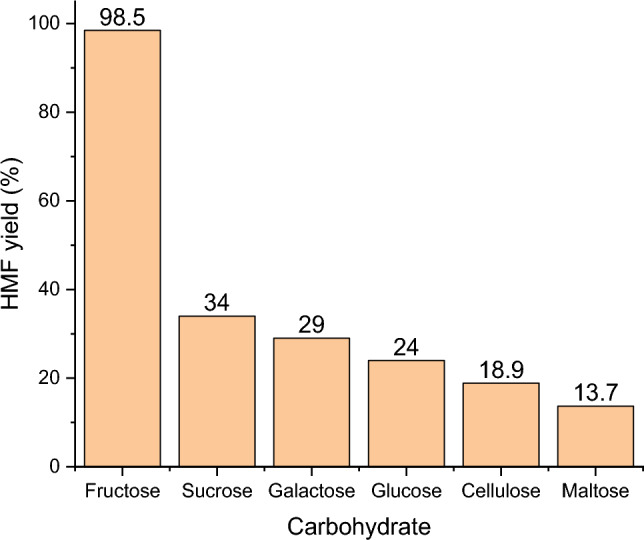
Catalytic activity of Hal-IL for conversion of various carbohydrates to HMF. Reaction conditions: catalyst loading 30 wt%, temperature 100 °C, time 100 min.

Investigation of the catalytic activity and recyclability of Hal-IL approved that this bio-based catalyst can be considered as a potential catalyst for promoting dehydration of fructose to HMF. To appraise the performance of this catalyst for the conversion of other carbohydrates to HMF, conversion of maltose, glucose, galactose, sucrose and callouses to HMF under the optimum reaction conditions was also examined. According to the results, Fig. 4, the examined carbohydrates resulted in low to moderate yields of HMF. More precisely, the second best substrate for the synthesis of HMF was sucrose that is composed of glucose and fructose units. In fact, the reaction of this substrate requires hydrolysis and isomerization of glucose to fructose followed by dehydration to HMF. Hence, this reaction is more complicated compared to the conversion of fructose that needs only dehydration step. Similarly, the reaction yields for maltose that is a disaccharide formed from two units of glucose and cellulose that consists of glucose units is even lower than that of sucrose.

### Reaction mechanism

**Figure 5:**
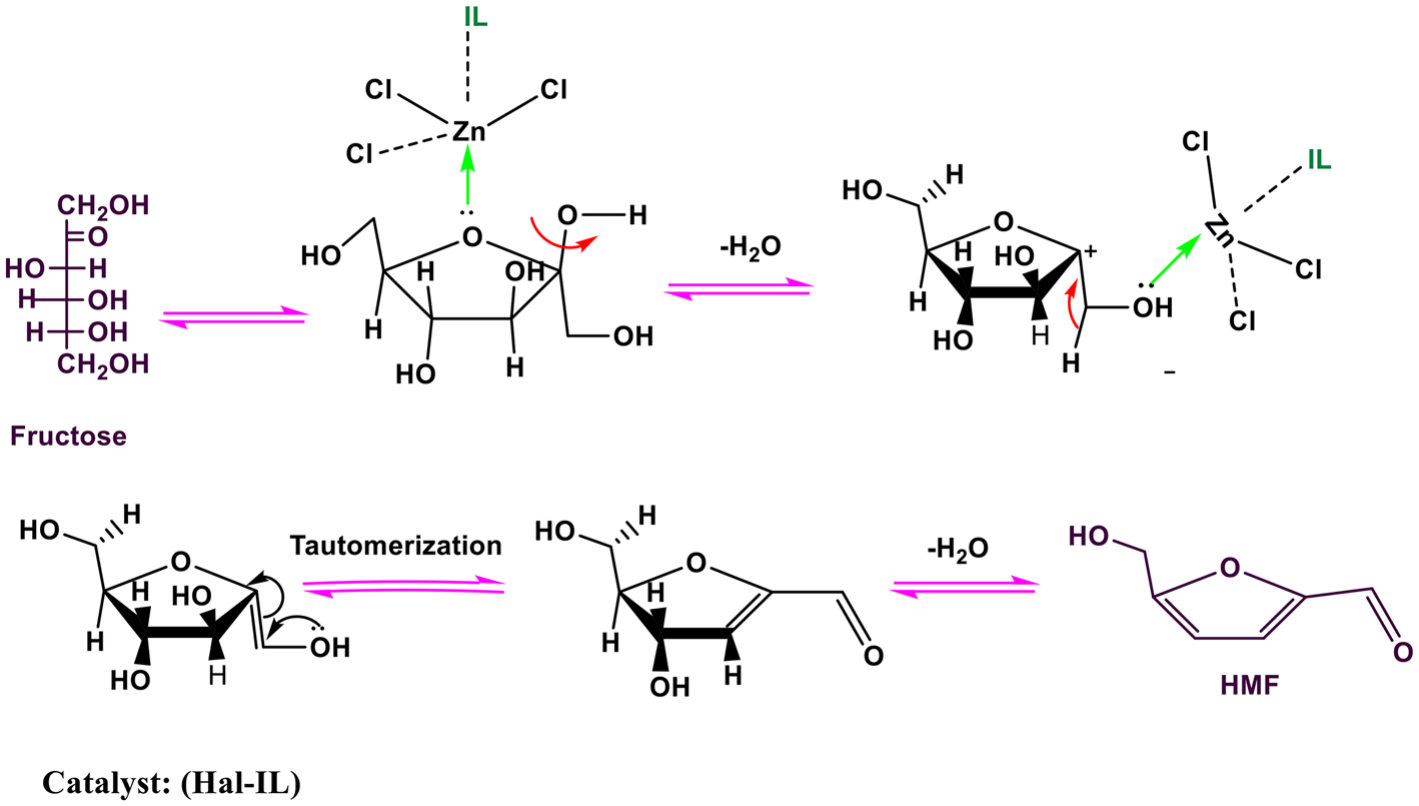
Proposed mechanism of conversion of fructose to HMF in the presence of Hal-IL.

The plausible mechanism of formation of HMF in the presence of Hal-IL is presented in Fig. 5. Adsorption of sugar on the surface of the catalyst can bring the substrate (sugar) in the vicinity of the main catalytic sites, i.e. acidic IL. As shown, the reaction proceeds by activation of fructose through coordination of the catalyst. Then, water removal forms an enolic intermediate, which tautomerize to form keto-form. Subsequently, loss of the second molecule of water occurs to furnish HMF.

### Kinetic study

In kinetic studies, temperature is an influential factor in the reaction rate constant. Fructose has been converted to HMF through a first-order process. The reaction rate is determined using Eq. (2). The reaction rate (r) demonstrates how fructose is consumed and 5-hydroxymethylfurfural is produced over time.

2 $$- {\text{r}}\left[ {{\text{fructose}}} \right] \, = \, \left( {{\text{d}}\left[ {{\text{HMF}}} \right]} \right)/\left( {{\text{dt}}} \right) \, = \, \left( { - {\text{d}}\left[ {{\text{fructose}}} \right]} \right)/\left( {{\text{dt}}} \right) \, = {\text{ K}}\left[ {{\text{fructose}}} \right].$$

If we consider the conversion of fructose, we can obtain Eq. (3):

3 $$\left[ {{\text{fructose}}} \right] \, = \, \left[ {{\text{fructose}}} \right] \cdot \left[ {{1} - {\text{x}}} \right].$$

Equation (4) is derived from integrating the above Eqs. (2) and (3). By using Eq. (4) and conducting experiments at four different temperatures, we can plot the − ln(1 − x) diagram against time for each temperature. The slope obtained from each linear plot represents the rate constant.

4 $$-{\text{ln}}\left(1-X\right)=kt+C.$$

The − ln(1 − X) term is used because it conveniently relates to the extent of conversion. The term (1 − X) represents the fraction of fructose that has not been converted, so ln(1 − X) gives us a measure of the remaining unconverted fructose. Taking the negative of ln(1 − X) allows us to plot the quantity that decreases as the reaction progresses.

In Eq. (4), “k” represents the rate constant, “t” is the time, and “C” is the intercept of the linear plot. The slope of the line, represented by “k”, provides valuable information about the rate at which the reaction proceeds.

By analyzing the − ln(1 − x) vs. time plots at different temperatures, we can determine how the rate constant varies with temperature and gain insights into the temperature dependence of the reaction kinetics.

**Figure 6:**
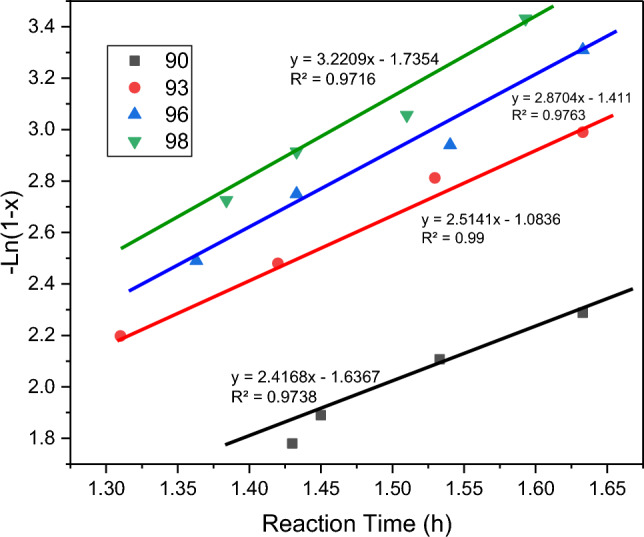
Amount of the rate constant value, K, at 4 different temperature.

According to Fig. 6, the rate constant value increases with increasing temperature. So from temperature with temperature values of 90, 93, 96, and 98 °C. The rate constant values were obtained as 2.4168, 2.51141, 2.870 and 3.2209, respectively. The observed results confirmed that the rate constant directly relates to temperature. According to the Arrhenius equation, Eq. (5), we can also calculate the activation energy.

5 $${\text{ln}}k=-\frac{Ea}{RT}+{\text{ln}}A.$$

In this equation, A is the pre-exponential factor or frequency factor, R is the universal gas constant, and T is the absolute temperature in Kelvin.

**Figure 7:**
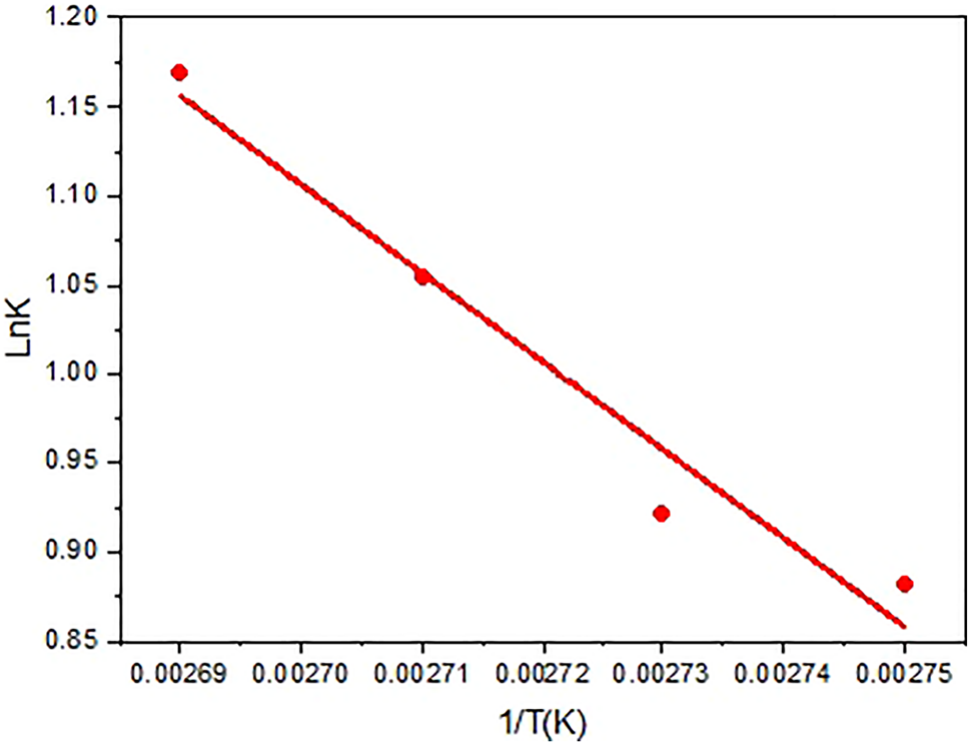
Calculating the amount of E_a_ for dehydration of fructose to 5-HMF.

When we plot ln k against 1000/T, a linear relationship is observed, Fig. 7, and the slope of the resulting line is equal to (− Ea/R). Therefore, by knowing the slope and the value of R, we can calculate the activation energy of the reaction. R is a fundamental constant in nature that relates the energy scale of a system to its temperature and the number of particles present.

According to this calculation, the Ea value was achieved as 22.85 kJ/mol as shown in (Fig. 7).

### Comparative study

As HMF synthesis is an important and essential reaction for the synthesis of furan-based biofuels and chemicals, many researchers attempted to disclose efficient catalysts for catalyzing this reaction. In this regard, various heterogeneous catalysts based on natural and synthetic supports have been developed, Table 3. Notably, synthesis of some synthetic supports, such as SBA and MCM need time-consuming procedures. As most of these porous supports are prepared under hydrothermal conditions, they are less cost-effective compared to natural supports. Noteworthy, in some synthetic porous supports, the size of the pores are so small that hindered efficient mass transfer. On the other hand, synthesis of some synthetic supports, such as metal organic frameworks, MOFs, require use of organic solvents, which makes them less environmentally benign.

**Table 3:** Comparison of catalysts, reaction conditions, and yields for the selective conversion of fructose to 5-HMF.

Entry	Catalyst (g)	Solvent	Temp. (°C)	Time (min)	HMF yield%	Ref.
1	Nb_2_O_5_	DMSO	120	120	86.2	^43^
2	Fe_3_O_4_@SiO_2_-SO_3_H	DMSO	100	120	93.1	^44^
3	MCM-41-PrSO_3_H	Water/nitromethane	140	30	59.52	^45^
4	SBA-15-Pr-SO_3_H	Water/nitromethane	140	30	69.75	^45^
5	Al-MCM-41	DMSO	165	30	59.4	^46^
6	Sulfated zirconia	Acetone/DMSO	180	20	72.8	^47^
7	MIL-101(Cr)-SO_3_H	DMSO	120	60	90	^48^
8	L-Proline-derived IL	-	90	50	73.6	^49^
9	Hal-IL	DMSO	100	100	98.5	This work

Compared to homogeneous catalysts, such as homogeneous ILs, use of heterogeneous counterparts is more favorable due to the facilitated recovery and more efficient recycling.

In conclusion, various catalysts reported for the dehydration of fructose to HMF have their own pros and cons and as the reaction conditions for each reaction is unique and distinguished from other reactions, precise comparison is not possible. However, from the data gathered in Table 3 it can be deduced that Hal-IL showed a reasonable and acceptable catalytic performance for the HMF synthesis. Notably, the merits of Hal-IL are the availability of its components (Hal and caffeine), facile synthetic procedure, simple recovery and efficient recyclability.

## Conclusion

A novel bio-based Lewis acid heterogeneous catalyst, Hal-IL, was designed and synthesized by using Hal as a natural support and caffeine as a bio-based imidazole source. More specifically, Hal was first covalently functionalized with TCT and then reacted with caffeine and ZnCl_2_ to produce Lewis acid IL on Hal. The role of TCT was providing more reaction sites with caffeine and increasing the number of grafted caffeine. The resultant catalyst was characterized and utilized for the synthesis of HMF from dehydration of fructose. RSM was applied to optimize the reaction conditions and investigate the effects of the reaction variables on the yield of HMF. It was found that using 30 wt% Hal-IL at 100 °C, HMF was achieved in 98.5% yield in 100 min. The recyclability of the catalyst showed that it could be recovered and recycled for at least six runs. The stability of the reused catalyst was also confirmed by its characterization. Notably, the catalyst exhibited higher catalytic activity than Hal and the control catalyst with only one caffeine-based ionic liquid, confirming the role of TCT and number of grafted ILs in the catalytic activity of the catalyst. According to the kinetic studies, the activation energy was estimated to be 22.85 kJ/mol.

## Experimental

### Materials

Hal (provided from China), 3-(amino propyl)-triethoxysilane (APTES, 99%), 2,4,6-trichloro-1,3,5-triazine (TCT, 99%), 1,3,7-trimethylxanthine (Caffeine, 99%), HMF, tetrahydrofuran (THF), potassium carbonate (K_2_CO_3_), dichloromethane (CH_2_Cl_2_), zinc chloride (ZnCl_2_ > 98%), fructose (> 99%), cellulose (> 99%), glucose (> 99%), maltose and, sucrose, anhydrous ethanol (> 99%), dimethyl sulfoxide (DMSO, > 99%), toluene were purchased from Sigma-Aldrich and used for the preparation of the catalyst and synthesis of HMF.

### Synthesis of Hall functionalized with caffeine-based ionic liquid: Hal-IL

The synthesis of the catalyst was conducted in four steps, which are elaborated in the following:

### Functionalization of Hal with APTES: synthesis of Hal-APTES

Hal (4 g) was suspended in dry toluene (60 mL) and stirred for 20 min at room temperature to give a homogeneous suspension. Then, APTES (4.5 mL) was added to the suspension and it was refluxed overnight under Ar atmosphere. The solid was then separated, washed with toluene, and dried at 60 °C for 24 h. Noteworthy, APTES was used to provide amino functional group on Hal and allow covalent growth of IL on its surface.

### Conjugation of TCT, synthesis of Hal-APTES-TCT

TCT (4.5 g) was dissolved in THF (30 mL) and added drop by drop to the suspension of Hal-APTES in THF (50 mL) in an ice bath at 0 °C. The mixture was stirred overnight and then the obtained precipitate was separated via centrifugation. The product, Hal-APTES-TCT was subsequently washed four times with THF and dried at 60 °C for 24 h.

### Reaction with caffeine: synthesis Hal-APTES-TCT-Caff

Caffeine (4.5 g) was dissolved in DMSO (20 mL) and introduced to the stirring mixture of Hal-APTES-TCT (6 g) in DMSO. The resultant mixture was stirred at 80 °C for 48 h under argon atmosphere. At the end of the reaction, the solid was collected, rinsed with THF and dried at 70 °C for 24 h.

### Reaction with ZnCl_2_: synthesis Hal-IL

To a stirring suspension of Hal-APTES-TCT-Caff (6 g) in THF (20 mL) ZnCl_2_ (9 g) was added and the mixture was stirred for 48 h at 25 °C. Then, the precipitate, Hal-IL, was separated, washed with CH_2_Cl_2_ several times, and dried at 80 °C overnight, Scheme 1.

### Synthesis of the control catalyst: Hal-IL1

Hal-IL1 control catalyst with only one caffeine-based IL was also synthesized as a control catalyst. To this purpose, Hal was first Cl-functionalized and then reacted with caffeine and ZnCl_2_ respectively, Scheme S1. The detail of the procedures for the synthesis of Hal-IL1 is similar to that of Hal-IL, except, CPTES was used instead of APTES. Moreover, the step for conjugation of TCT was omitted.

### Characterization of the catalyst

The details of the used apparatus and devices for the characterization of Hal-IL are listed in Supporting Information.

### Conversion of fructose to HMF

To convert fructose to HMF, to a solution of fructose (0.1 g) in anhydrous DMSO (4 mL) Hal-IL (0.03 g) was added and the mixture was stirred at 100 °C for 100 min. Then, Hal-IL was separated, washed several times with DMSO and dried at 50 °C for 24 h.

### Measurement of HMF yield

To calculate HMF yield, Eq. (6) was employed, where, Mole (I) is the initial moles of fructose.

6 $$HMF\, yield \left(\%\right)=\frac{Mole (HMF)}{Mole\left(I\right)}\times 100\% .$$

Notably, high-performance liquid chromatography (HPLC) and Gas chromatography (GC) were applied for the quantitative analyses.

To conduct HPLC analysis, Agilent 1200 Series device equipped with a Brisa LC2 C18 column (5 µm, 25 $\times$ 0.46) operated at 35 °C based on the external standard was utilized. In this analysis, pure HMF was used and its retention time was compared with the sampler.

For GC, Agilent 6890 device with a flame ionization detector (FID) and G&W HP-5 ms GC column was employed.

### Supplementary Information

Supplementary Information.

## Data Availability

The datasets used and/or analyzed during the current study are available from the corresponding author on reasonable request.
