# Supplementary material for: Clay-supported bio-based Lewis acid ionic liquid as a potent catalyst for the dehydration of fructose to 5-hydroxymthylfurfural
Source: Sci Rep. 2024 Jan 2;14:82. doi: 10.1038/s41598-023-50773-2 (PMC10762215; doi:10.1038/s41598-023-50773-2)
Supplement: Supplementary file 1 — Supplementary Information. [file 41598_2023_50773_MOESM1_ESM.docx]

**Supporting information**

**Clay-supported bio-based Lewis acid** **ionic liquid as a potent catalyst for the dehydration of fructose to 5-hydroxymthylfurfural**

Soheila Yaghoubi^1^, Samahe Sadjadi^2*^, Xuemin Zhong^3^, Peng Yuan^4^**,** Majid M. Heravi*^1^

**Characterization of the catalyst**

Thermogravimetric analysis (TGA, METTLER TOLEDO, under O_2_ atmosphere and ramp rate of 10 °C/min) was applied to study the thermal stability of Hal-IL and estimating the loading of IL on Hal. The structure and crystalline phase of Hal and Hal-IL were investigated using X-ray diffraction (XRD, Rigaku Ultima $Ⅳ$ with Cu-Kα). Conjugation of IL on Hal was confirmed using Fourier-Infrared spectroscopy (FTIR, BRUKER TENSOR 35 spectrophotometer 65 with a scan time of 1s and spectral resolution of 2 cm^-1^ by using potassium bromide (KBr) pellets). The specific surface area (S_BET_) of the catalyst was measured using Brunauer-Emmett-Teller (BET) method employing BELSORP MINI II, BEL instrument with pre-heating at 150 °C for 3 h. The morphological study was conducted using Scanning electron microscope (SEM) coupled with energy-dispersive X-ray spectroscopy (EDS, VEGAII TESCAN device, equipped with QX2, RONTEC energy dispersive X-ray analyzer).

**Scheme** **S1**. Schematic synthetic route of Hal-IL1 control catalyst.


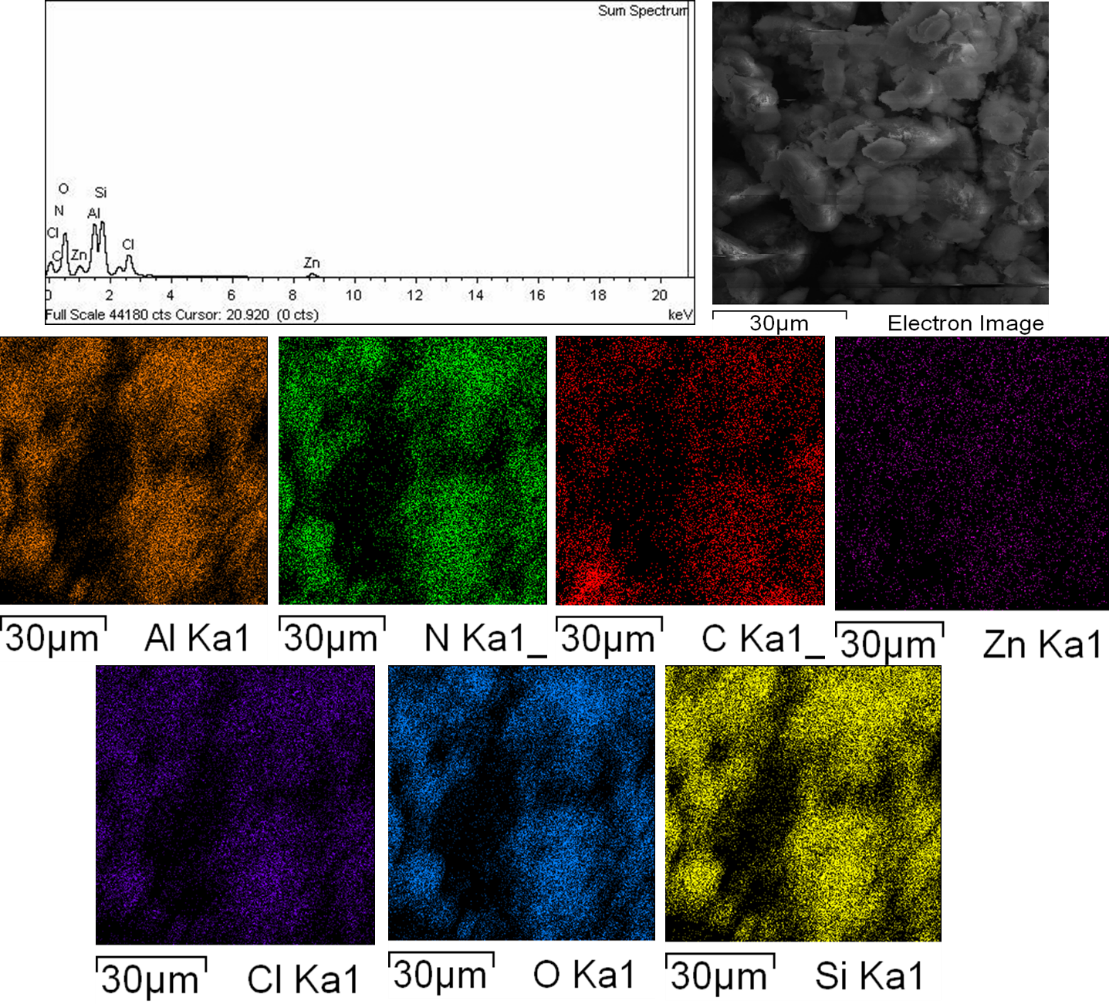


**Figure S1.** EDS and elemental mapping analysis of Hal-IL.

**Figure S2.** Nitrogen adsorption-desorption isotherm of Hal-IL
